# The current state of paediatric intestinal transplantation: A global review

**DOI:** 10.1016/j.intf.2025.100334

**Published:** 2025-12-02

**Authors:** Sinead Cunningham, Jonathan Hind

**Affiliations:** Department of Paediatric Gastroenterology, Hepatology and Nutrition, Kings College Hospital NHS Foundation Trust, London, United Kingdom

**Keywords:** Intestinal Failure (IF), Parenteral Nutrition (PN), Intestinal Transplant (ITx), Intestinal Rehabilitation (IR), Intestinal Transplant Registry (ITR)

## Abstract

Paediatric intestinal transplantation (ITx) has evolved from a high-risk experimental procedure into a life-saving therapy for children who have complications of intestinal failure (IF) or who cannot be stabilized by nutritional rehabilitation. Indications include complications of irreversible intestinal failure from short bowel syndrome, motility disorders, or intestinal dysplasia, with graft options ranging from isolated intestine to liver-intestine or multivisceral transplantation, including reduced-size and living donor grafts. Advances in surgical techniques, perioperative care, and immunosuppression have markedly improved survival and functional outcomes, although early and late graft loss, rejection, and infectious complications remain important challenges. Optimized immunosuppression protocols have reduced acute rejection rates, but also facilitated successful retransplantation when required. Multidisciplinary management addressing growth, neurodevelopment, and psychosocial well-being is recognized as being essential for improving long-term quality of life. Despite these advances, significant global disparities persist in access to paediatric ITx, driven by donor scarcity, financial constraints, and limited expertise in low-resource regions. Collaborative networks, telemedicine platforms, and international registries have emerged to share knowledge, standardize care, and support capacity-building. Looking forward, advancements in immunosuppression, expanded living donor programmes and international collaboration hold promise for further improving outcomes and broadening access. Increasing recognition of the benefits of earlier referral and transplantation is shifting the paradigm from viewing ITx solely as a rescue therapy toward proactive management to optimize survival, growth, and quality of life. Collectively, these developments indicate a future in which paediatric ITx offers not only life-saving treatment but also enhanced long-term health, improved quality of life, and equitable access for children worldwide.

## Introduction

Intestinal failure (IF) may be defined as insufficient functional gut mass to maintain absorption of nutrients and fluids necessary for normal growth and development [1]. In children, short bowel syndrome (SBS) is the leading aetiology, followed by motility disorders and congenital or acquired mucosal enteropathies [2]. Long-term parenteral nutrition (PN) revolutionised survival since the late 1960s, yet historical outcomes were poor, with 2–5-year mortality rates approaching 40 % due to sepsis, liver failure, and loss of venous access [3]. The emergence of multidisciplinary intestinal rehabilitation (IR) programmes in the 1990s transformed care, integrating medical, surgical, and nutritional strategies to promote intestinal adaptation and reduce PN complications. Advances in catheter care, lipid emulsions, bowel-lengthening procedures, and GLP-2 analogues have further improved outcomes, with long-term PN survival now exceeding 80 % in specialised centres [4], [5], [6].

Nevertheless, enteral autonomy is not universal; large multicentre cohorts report that ∼40–55 % of patients achieve autonomy [6], selected SBS cohorts report ∼70–76 % [7]. If enteral autonomy is not achieved, IF-associated complications remain major determinants of prognosis and health-related quality of life (HRQoL). Catheter-related bloodstream infections (CLABSI), venous thrombosis with loss of access, and intestinal-failure associated liver disease (IFALD) continue to affect many children- approximately 10–20 %, which can be severe [8], [9]. Consequently, intestinal transplantation (ITx) remains a life-saving intervention [10]. Recently, referral criteria have expanded. Beyond physiological indicators, current guidelines recognize significantly impaired HRQoL, psychosocial distress, and caregiver exhaustion as legitimate grounds for transplant consideration [11], [12].

## History of ITx

Historically, outcomes for paediatric ITx were poor. Early transplants in the 1960s and 1970s were largely unsuccessful due to overwhelming rejection and infection. The first successful isolated small bowel transplant in a child was reported in the late 1980s, establishing technical feasibility and marking a turning point in the field [5]. Around the same time, the introduction of tacrolimus significantly improved survival [13], initiating consistent paediatric transplant success.

Introduction of multidisciplinary IR programs in the late 1990s marked a paradigm shift. By optimizing PN, promoting intestinal adaptation, and reducing complications, IR programmes allowed more children to remain stable on PN, thereby reducing the need and urgency of transplantation for many [4], [7].

By the early 2000s, advances in perioperative care and advances in immunosuppression, such as the adoption of anti-thymocyte globulin (ATG) for induction immunosuppression in some centres, helped raise one-year ITx survival rates above 70 % [4], [14].

As outcomes of isolated ITx improved, more complex procedures became feasible. Multivisceral transplants (MVT) were used for children with advanced IFALD or complex abdominal pathologies, such as severe dysmotility syndromes or long-segment Hirschsprung’s disease [15], [16]. Graft design has become increasingly individualized, with organs added or excluded based on comorbidities. A notable advancement has been the selective inclusion of the colon in grafts. Initially approached cautiously due to infection concerns, recent evidence demonstrates physiological and functional benefits, with capacity to increase likelihood of enteral autonomy thanks to the absorptive functions of the colon and the production of GLP-2, without increased infection risk. The proportion of colon-containing grafts rose from 4 % in 2000 to 30 % by 2012, reflecting growing clinical acceptance and refined surgical expertise [17], [18], [19]. This trend exemplifies a patient-centred approach to graft selection aimed at optimizing post-transplant HRQoL.

Improvements in immunosuppressive protocols, central line care, and perioperative management have reduced infection-related mortality. High-volume centres now report long-term survival exceeding 80 %, with enteral autonomy achieved in up to 75 % of patients, depending on the cohort [6].

Despite these, ITx remains one of the least frequently performed solid-organ transplants, with global annual volumes below 200 procedures, roughly half of which are in paediatric recipients [16]. This is due to a combination of surgical complexity, high resource requirements, and limited number of specialised centres [20]. Although graft survival has improved [14], waitlist mortality remains high [8], highlighting the importance of timely referral. The modern approach prioritizes earlier intervention, supported by IR programmes, and guided not only by clinical deterioration but also by HRQoL, caregiver well-being, and developmental potential [4], [7].

## Indications for intestinal transplantation

A comparison of the position papers from Kaufman et al. (Table 1) highlights a paradigm shift in the approach to ITx. Early guidance in 2001 emphasised ITx as a last resort, reserved for children with irreversible IF who developed life-threatening PN complications such as end-stage IFALD, recurrent CLABSI, or loss of venous access. In contrast, the 2019 consensus paper reflected a proactive, individualised philosophy: referral should occur earlier, with careful consideration of growth, neurodevelopment, HRQoL and family burden alongside traditional medical criteria. This evolution represents a move away from salvage therapy toward preventing irreversible morbidity and preserving long-term outcomes.

**Table 1:** Comparison of the 2001 and 2019 position papers on indications for intestinal transplant [11], [12].

**Category**	**2001 Kaufman et al.**	**2019 Kaufman et al.**
**Overall philosophy**	Focused on preventing mortality from PN-related complications.	Early referral; proactive, individualised care balancing PN risks with ITx outcomes.
**Main indications**	Severe PN complications: loss of venous access, recurrent sepsis, advanced IFALD, intractable dehydration.	Broader: failure of intestinal rehabilitation, severe metabolic bone disease, persistent metabolic instability
**Liver disease**	Combined liver–intestinal transplant indicated for advanced cholestatic liver disease.	Earlier intervention; detailed staging to support transplant before irreversible IFALD.
**Growth & development**	Growth failure less emphasised.	Failure to thrive, impaired growth, and neurodevelopmental risk considered key indications.
**PN failure vs. adaptation**	Emphasis on PN failure.	Greater focus on assessing intestinal adaptation and maximising rehabilitation before ITx.
**Infection & sepsis**	Recurrent CLABSI central indication.	Still important, but emphasis on optimising catheter care prior
**Timing of referral**	Typically late, once catastrophic complications emerged	Strong focus on early referral to specialised centres for comprehensive evaluation.

Building on these developments, subsequent discussions by members of the International Intestinal Rehabilitation and Transplant Association (IIRTA), presented at the 2025 Congress of the International Intestinal Rehabilitation and Transplant Association (CIIRTA), recommended further expansion of referral criteria. Suggested considerations now include: ultrashort gut (<20–25 cm residual small bowel in neonates), total intestinal aganglionosis, persistent intestinal pseudo-obstruction unresponsive to enterostomy, patients without safe access to home PN (e.g. due to cost or health system limitations), and early-stage IFALD before progression to advanced, irreversible liver injury.

These recommendations reflect the recognition that ITx should not be delayed until catastrophic PN complications occur (thus also adversely affecting post-ITx outcomes), but should be considered when medical, nutritional, and psychosocial burdens threaten long-term outcomes [9], [21].

It is important to acknowledge that access to home PN, IR programs, GLP-2 analogues, and ITx remain highly variable worldwide. In many low- and middle-income countries, therapies such as home PN are not universally available, meaning “ideal” referral criteria cannot always be applied. In these contexts, transplant may be considered earlier, if PN cannot be sustained safely at home. Studies on timing [22] and cost [23] have largely been conducted in North American settings and therefore do not account for these disparities. More recent international consensus statements emphasise that indications must be adapted pragmatically to reflect healthcare infrastructure and resources [9], [21], [24], [25]. This global disparity underscores the need for locally-specific guidelines, adapted to the resources and realities of each healthcare system.

## Types of graft

**Fig. 1:**
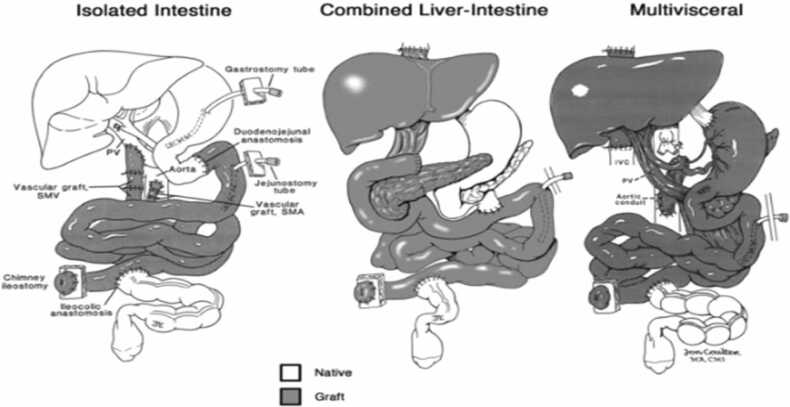
Schematic representation of intestinal transplant graft types [26].

Once a child is deemed a candidate for ITx, graft selection is tailored to the underlying pathology, the presence and severity of IFALD, and prior surgical history. While nomenclature varies across centres, four main graft types are recognised (Fig. 1):

1. Isolated small bowel transplant: For children with irreversible IF but preserved liver function
2. Combined liver–small bowel transplant: Indicated when IFALD has progressed to advanced or irreversible liver disease
3. Multivisceral transplant (MVT): Includes stomach, pancreas, intestine, and liver, typically reserved for complex conditions such as severe dysmotility syndromes, Hirschsprung’s disease, or intra-abdominal tumours.
4. Modified multivisceral transplant (MMVT): Similar to MVT but without the liver, used when hepatic function remains adequate

Graft design is highly adaptable, with organ inclusion or exclusion determined by patient-specific comorbidities. For example, the colon may be added to optimise gastrointestinal function, and combined liver-small bowel-kidney grafts are occasionally performed in children with concomitant renal failure. This flexibility underscores the individualised nature of graft planning in paediatric ITx.

## Global activity and access

In the modern era, paediatric ITx has expanded globally, though access remains inequitable. Registries such as the International Intestinal Transplant Registry (IITR) and the International Intestinal Failure Registry (IIFR) play a central role in tracking global activity, procedural types, outcomes, and regional disparities, guiding contemporary consensus on indications, timing, and graft selection [15], [27].

The IITR, established in 1985, has tracked global paediatric ITx activity, procedural types, and accessibility [15], [20]. From 1985–2025, a total of 2692 paediatric intestinal transplants were performed across 76 centres worldwide. Of these, 780 (29 %) were isolated small bowel transplants, 53 were MMVT, and the majority were liver-inclusive transplants, comprising 1022 liver–intestine and 511 MVT [20]. Analyses indicate a gradual decline in annual paediatric ITx procedures, likely reflecting advances in medical and surgical therapies for IR- including GLP-2 analogues, surgical bowel-lengthening techniques, and optimized PN- that have improved survival and decreased overall need for transplantation [28].

**Fig. 2:**
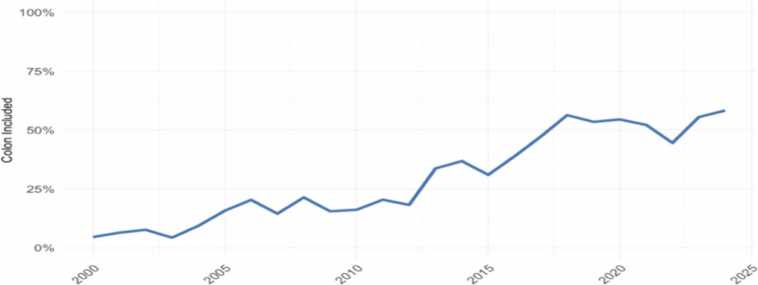
Inclusion of colon in graft 2000–2025. Combined adult and paediatric data [20].

A notable change over time has been the increasing colon inclusion in grafts to increase chances of enteral autonomy, with combined adult and paediatric data suggesting up to 50 % colon inclusion in grafts *(*Fig. 2*).*

**Fig. 3:**
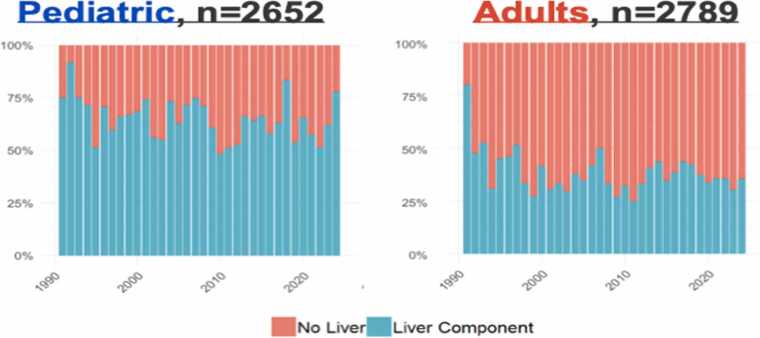
Increased liver containing grafts in paediatric cohort compared to adult populations [20].

In contrast to adults, the higher proportion of liver-containing intestinal grafts in children, may reflect late referral with established IFALD, although improved outcomes with combined grafts have also supported their wider use (Fig. 3)*.*

Access and outcomes vary substantially across regions, reflecting differences in healthcare infrastructure, centre expertise, and economic resources. North America hosts several high-volume centres with comprehensive multidisciplinary programs. Registry data and the United Network for Organ Sharing (UNOS) report one-year survival exceeding 85 %, with five-year survival around 70 % [29], comparable to outcomes of other high-risk solid organ transplants. In Europe, outcomes are broadly similar, with one-year survival typically 80–85 % and five-year survival 65–70 %, depending on centre and country [11], [30], [31].

In Asia, paediatric ITx remains relatively underdeveloped compared to Western regions. Japan and South Korea have established advanced national programs, including living donor small bowel transplantation, achieving one-year survival rates of approximately 80 % [32]. Japan has maintained an ITx program since 1996, supported and covered by the national healthcare system. Singapore has more recently initiated its own paediatric ITx program. In contrast, many other countries in the region continue to encounter substantial barriers, including limited institutional expertise, high procedural and post-operative costs, delayed referral pathways, inconsistent availability of immunosuppressive agents, and variable standards of post-transplant follow-up. Nonetheless, isolated centres in China and India have reported favourable outcomes, and increasing regional collaboration and knowledge exchange are gradually addressing disparities in access and outcomes across Asia [33], [34]

In many parts of the Middle East, access to home PN and structured IR programmes remains scarce, leading to sometimes unavoidable reliance on transplantation. Recent reports from Iran and the wider region describe the largest experience of paediatric and adult ITx in a setting without home PN, underscoring the unique challenges of patient selection, postoperative management, and long-term survival in resource-limited healthcare systems [35], [36], [37]. In Saudi Arabia, an ITx unit was set up in 2017, with adult 1 and 5-year patient survivals of 91 and 64 % respectively [38].

Latin America continues to have limited access to paediatric ITx, with few specialized centres. One-year survival ranges from 70 to 80 %, though long-term outcomes are lower due to challenges in managing complications, medication shortages, and limited follow-up infrastructure. These studies highlight how regional constraints shape clinical decision-making and contribute to disparities in access and outcomes [39].

Registry data underscore persistent disparities in access. Many children in resource-limited settings cannot access home PN, multidisciplinary IR programs, or specialized transplant centres. In such contexts, referral criteria may be adapted: transplantation may be delayed due to insufficient infrastructure, or pursued earlier when prolonged PN is unsafe [40], [41], [42]. Registries therefore remain crucial for documenting these inequities, guiding resource allocation, and informing context-specific guidelines [27].

## Financial and access considerations

The long-term management of paediatric IF with PN carries a substantial financial burden. In specialist tertiary centres in the United Kingdom, patient-level analyses report first-year costs for children with SBS can exceed GBP £ 90,000–120,000, reflecting hospitalization, catheter care, PN solutions, and outpatient services [43]. In the United States, first-year costs are estimated at US $120,000–180,000, primarily driven by central venous catheter placement, CLABSIs, and prolonged hospitalization [44]. Annual outpatient expenses—including PN solutions, lipid emulsions, and infusion supplies—range from US $50,000 to US $80,000, with indirect costs such as home nursing support and caregiver income loss adding an additional US $25,000–40,000 per year [44].

In contrast, ITx involves a high initial expenditure—approximately US $500,000–850,000—accounting for graft procurement, surgical intervention, intensive care, and induction immunosuppression [45]. However, ongoing costs decline markedly post-transplant, averaging US $30,000–50,000 annually for maintenance immunosuppression, monitoring, and episodic care [23].

Over a five-year period, total expenditures for both PN and ITx converge, with cumulative costs ranging from US $700,000–1.1 million for PN and US $750,000–1.0 million for ITx [46]. Cost-effectiveness analyses indicate a break-even point within 6–8 years—sooner in patients who achieve enteral autonomy after transplant [47]. When quality-adjusted life years (QALYs) are factored in, ITx yields an incremental cost-effectiveness ratio (ICER) of US $180,000–240,000 per QALY. While this is near the upper boundary of U.S. cost-effectiveness thresholds, it becomes more favourable when broader developmental, psychosocial, and societal benefits are considered [23].

Families also bear additional indirect costs, including travel, accommodation, income loss, and psychosocial stress—factors that are frequently excluded from formal economic assessments but substantially impact the overall burden of care [40].

These financial considerations directly influence real-world timing of referral, graft selection, and post-transplant outcomes, highlighting that survival, enteral autonomy, and complication rates are closely linked to the economic burden of care.

## Survival and complications

Paediatric ITx outcomes have continued to improve**.** According to the 2025 IITR update, overall paediatric graft survival remains at about 70 % at 1 year and 51 % at 5 years for all transplants performed during 1985–2025. For patient survival, IITR reports 76 % at 1 year and 60 % at 5 years in the paediatric population across all eras [20]. The IITR remains the most comprehensive global source of intestinal transplant data, though, as with many registries, variations in reporting and external validation over time should be acknowledged when interpreting outcomes.

When focusing on more recent transplant eras (post-2000 / post-2015), outcomes are better: single-centre and registry data suggest 1-year graft survival rates in the 75–85 % range and up to 65 % at 5 years.

Graft and patient outcomes still vary by graft type and underlying diagnosis [48], [49]. Isolated small bowel grafts tend to fare better in the early post-transplant period compared to liver-inclusive or multivisceral grafts, which may reflect the severity of underlying liver disease [50].

The underlying diagnosis of IF (e.g. short bowel syndrome vs motility disorder or congenital enteropathy) also continues to influence prognosis, with SBS generally associated with more favourable outcomes [50].

**Fig. 4:**
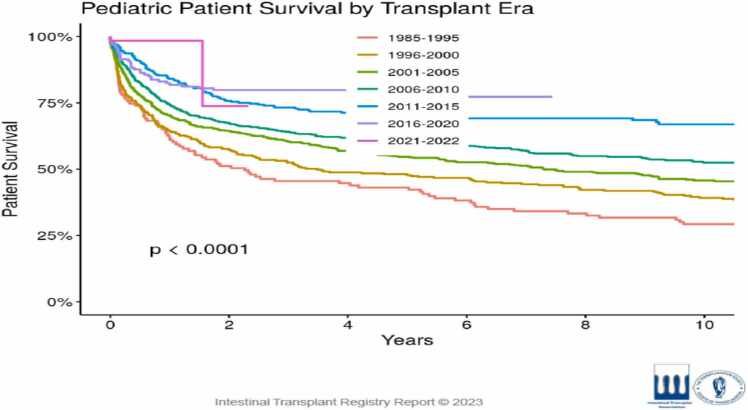
Paediatric patient survival by transplant era [20].

Thus, the most contemporary data demonstrate that 1-year outcomes in paediatric ITx are now > 80 %- and registry median outcomes support that survival has steadily improved over time (Fig. 4)*.*

**Fig. 5:**
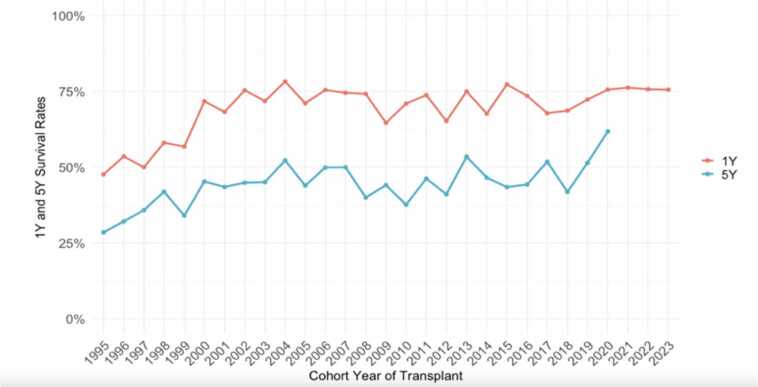
One and Five-year graft survival in paediatric intestinal transplant recipients over time, showing progressive improvement in both groups [20].

**Fig. 6:**
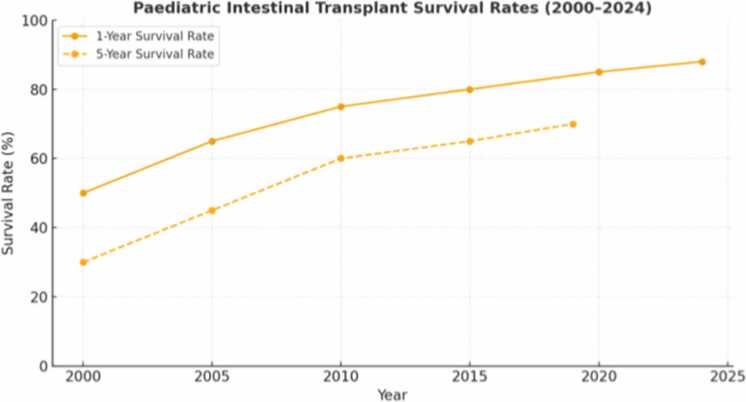
Five-year graft survival over time in paediatric and adult cohorts [20]. 5- year survival data shown to 2019 to reflect completed follow up.

Parallel improvements in graft survival have also been documented, with both paediatric and adult cohorts showing progressive gains in 5-year outcomes since the early 2000s (Fig. 5)*,* with paediatric results equalling or surpassing those in adults (Fig. 6).

Infectious complications and rejection remain leading causes of morbidity, though advances in antimicrobial prophylaxis, rejection surveillance, and multidisciplinary follow-up have progressively improved outcomes [42], [51]. Centre experience and the presence of comprehensive multidisciplinary programs remain critical determinants of long-term success [52].

Beyond graft survival, functional outcomes are also encouraging. Both registry and single-centre analyses report that 60–70 % of paediatric recipients achieve sustained enteral autonomy within 1–2 years post-transplant, permitting discontinuation of PN [52], [53]. Achievement of enteral independence is strongly associated with improved growth, neurodevelopment, and HRQoL, underscoring the functional value of ITx beyond survival alone [12].

Graft rejection and infection remain the leading causes of graft loss following paediatric ITx. Acute T-cell mediated rejection continues to be relatively common, occurring in approximately 25–35 % of recipients within the first post-transplant year [54], [55], [56]. Chronic rejection has become less frequent, now affecting roughly 5–10 % of patients, largely due to improved immunosuppressive protocols, early detection, and aggressive management strategies [55], [56].

Viral infections- particularly cytomegalovirus (CMV), Epstein–Barr virus (EBV**)**, and adenovirus- remain significant contributors to both graft dysfunction and patient morbidity, especially in paediatric recipients, who are often immunologically naïve [15], [57]. Despite advances in prophylaxis, antiviral therapy, and routine surveillance, these infections continue to account for a major proportion of early complications and remain important determinants of long-term outcome [58], [59].

Long-term complications extend beyond infection and rejection, encompassing graft-versus-host disease (GVHD), post-transplant lymphoproliferative disorder (PTLD), and cumulative toxicities of immunosuppression, including renal dysfunction and secondary malignancies [8], [60], [61], [62], [63], [64].

## Immunosuppression

Immunosuppression remains central to graft survival in paediatric ITx, and its evolution has markedly improved outcomes, reducing early graft loss and mortality. Early regimens in the 1980s relied on high-dose steroids and cyclosporine, with limited success due to high rejection and infection rates [65]. The introduction of tacrolimus in the 1990s was a major advance, improving 1-year graft survival from below 50 % to over 70 % [20] and allowing steroid minimization while reducing infectious complications [66].

Modern protocols emphasize personalized, risk-adapted therapy to balance rejection prevention with infection risk and long-term toxicity. Induction typically uses ATG or basiliximab, often with a short steroid course. Maintenance relies on tacrolimus, often combined with mycophenolate mofetil or mTOR inhibitors such as sirolimus, with dosing tailored to age, graft type, and immunologic risk. Liver-inclusive grafts confer an immunoprotective effect, permitting lower immunosuppression and reduced severe rejection compared to isolated small bowel grafts [67], [68].

Emerging strategies such as biologic therapies including eculizumab, target refractory or high-risk antibody-mediated rejection (AMR) [69], while gut-specific therapies like vedolizumab may reduce systemic immunosuppressive burden [70]. Genetic factors, such as NOD2/CARD15 variants, and immunogenetic profiling are increasingly integrated to tailor therapy to individual immune risk and graft characteristics [71]. These advances mark a shift toward personalized, precision-based immunosuppression in paediatric ITx.

## Donor specific antibodies

The development of DSAs, has emerged as a key immunological factor influencing graft survival and long-term outcomes in paediatric ITx. They are broadly classified into two categories: preformed DSAs (present before transplantation) and de novo DSAs (develop after transplantation). Both are associated with an increased risk of AMR, chronic rejection, and reduced graft survival [72].

Preformed DSAs due to prior sensitizing events are challenging in paediatric ITx due to the increased risk of early AMR and poor graft outcomes. According to Abu-Elmagd et al. [73], the presence of preformed DSAs in ITx correlates with lower long-term survival, especially in non-liver containing grafts [74]. The liver has a protective, immunomodulatory effect that mitigates DSA impact, a phenomenon not observed in isolated intestinal grafts.

De novo DSAs (dnDSAs) are associated with a significant risk of chronic rejection, late graft loss, and poorer overall survival compared to patients without DSAs [75], [76]. Graft injury, whether from repeated T cell–mediated rejection or from ischaemia–reperfusion injury, further increases the risk of dnDSA development.

Several studies have demonstrated that DSA-positive paediatric recipients have significantly reduced five-year graft survival compared to their DSA-negative counterparts [77].

Long-term graft survival is significantly improved when DSAs are detected and managed early. In highly sensitized patients, desensitisation strategies can reduce preformed antibody levels before transplantation. These typically include plasmapheresis or intravenous immunoglobulin (IVIG), and B-cell depletion using agents such as rituximab [78], [79], [80]. These strategies have been associated with reductions in DSA titres and improved engraftment outcomes among sensitized paediatric candidates [80].

Following transplantation, rescue strategies are employed when DSAs are newly detected or rebound despite initial desensitisation. Multimodal regimens incorporating rituximab (anti-CD20 monoclonal antibody for B-cell depletion), IVIG, plasmapheresis and eculizumab (a complement inhibitor), have demonstrated benefit in reducing AMR injury and preventing graft loss [78], [79], [80]. Importantly, outcomes are more favourable when these interventions are implemented promptly after DSA detection or evidence of AMR, rather than once chronic rejection is established.

Emerging strategies focus on precision immunosuppression, incorporating regular DSA monitoring and personalized therapies such as proteasome inhibitors (bortezomib) [79], [80]. Additionally, novel biomarkers for early detection of AMR are under investigation to improve outcomes further [73].

## Retransplantation in paediatric intestinal transplantation

Up to 10–20 % of paediatric ITx recipients will require retransplantation, most often because of chronic graft dysfunction, irreversible rejection, or recurrent infections after the initial transplant [81]. The decision to retransplant depends on several factors, including the patient's overall condition, the cause of graft failure, age, immunosuppression status, and donor availability.

Historically, survival rates after retransplantation were substantially worse than after primary transplant [82], but more contemporary reports demonstrate that outcomes are comparable in selected cohorts. Registry analyses and single-centre studies, including the 2020 series by Kara Balla et al., show 5- and 10-year patient survival rates that approach those of first transplants when retransplantation is performed in optimized candidates at experienced centres [83]. Long-term prognosis is influenced strongly by the cause of graft loss; outcomes are significantly worse when retransplantation is required for vascular thrombosis or chronic rejection, compared with cases of isolated ischemia or infection [79]. These findings underscore the importance of early diagnosis, timely referral, and tailored immunosuppression protocols to improve survival and achieve durable graft function.

## Psychological and quality-of-life considerations

In addition to traditional medical indicators, psychological well-being and HRQoL have become increasingly recognized as important factors in determining eligibility for paediatric ITx. Children reliant on long-term PN frequently endure significant limitations in their daily lives, including recurrent hospitalization, social isolation, growth delay, and reduced participation in normal childhood activities [84]. These challenges can lead to psychological distress, such as anxiety, depression; and impaired cognitive or emotional development [85]. Moreover, the burden on families- manifested through caregiver fatigue, disrupted family routines, and financial stress- can further exacerbate the psychosocial toll of chronic IF [86]. In such cases, where ongoing medical management fails to support an acceptable standard of living, transplantation may be considered as a means to restore functionality and improve overall well-being. This highlights the importance of a multidisciplinary approach that incorporates psychological assessment and patient-reported outcomes as part of the listing criteria for transplantation.

Given that HRQoL is an important factor in determining candidacy for paediatric ITx, it is important to consider the long-term psychosocial outcomes in these patients. Despite facing considerable medical challenges, many paediatric ITx recipients go on to achieve good educational attainment and maintain employment in adulthood, demonstrating meaningful functional recovery and successful social reintegration [87]. However, approximately one-third of paediatric ITx recipients experience mental health issues, including depression and substance use disorders such as alcohol and drug abuse, highlighting the ongoing vulnerability of this population [86]. These mental health challenges underscore the need for continued psychosocial support well beyond the immediate post-transplant period. Psychological monitoring and tailored interventions, including counselling and psychiatric care, are essential components of comprehensive long-term care to optimize quality of life and functional outcomes in paediatric ITx survivors.

## Future directions and the outlook for paediatric ITx

Future directions in paediatric ITx are increasingly shaped by progress in immunology, surgical innovation, and global collaboration. Immunological tolerance remains a central goal, with strategies including regulatory T-cell therapy, tolerogenic dendritic or mesenchymal stromal cell infusions [88], and costimulatory blockade with agents such as belatacept [89]. Equally important are advances in graft injury monitoring. Non-invasive biomarkers such as donor-derived cell-free DNA (dd-cfDNA), gene expression panels, and microRNA profiling may enable earlier detection of rejection and allow for earlier and more precise immunosuppression management [90].

The gut microbiome is also emerging as a modifiable determinant of transplant outcomes and potential therapeutic target, as dysbiosis can promote inflammation and alloimmune activation [90]. Future strategies such as probiotics, prebiotics, or faecal microbiota transplantation are being explored to restore microbial balance [91], [92]. On the surgical front, improved graft preservation techniques such as normothermic machine perfusion [93] and the expansion of living donor transplantation, particularly in infants and small children, have the potential to reduce wait times and increase access to appropriately sized grafts [94]. Looking further ahead, regenerative medicine holds transformative potential. Organoid and scaffold-based intestinal constructs are advancing in preclinical studies, although vascularisation and functional integration remain major hurdles [95]. Finally, collaboration and equity will be critical to progress. Telemedicine and international networks such as LIFT-ECHO, the IIRTA and its congress, along with the IIFR and IITR, provide platforms for knowledge-sharing, multicentre training, and reduction of global disparities [27], [55], [96].

## Conclusion

Paediatric ITx has progressed from a high-risk experimental intervention to an established, life-saving therapy with steadily improving outcomes. Once reserved as a rescue option, it is now recognized that earlier referral can preserve and improve growth, development, and HRQoL. Advances in immunosuppression and multidisciplinary care have reduced graft loss and enabled successful retransplantation. Yet, major challenges persist, including disparities in access, donor scarcity, and long-term complications. Increasing collaboration between intestinal rehabilitation and transplant programs, supported by knowledge-sharing networks and data from international registries is emerging as essential for timely referral, optimal patient selection, and avoidance of late, high-risk transplantation. The future lies not only in scientific innovation but also in global collaboration and equitable implementation, ensuring that the benefits of paediatric ITx extend to children in all healthcare settings.

## Ethical clearance

Not Applicable

## Patient's/ Guardian's consent

Not applicable.

## CRediT authorship contribution statement

**Jonathan Hind:** Writing – review & editing. **Sinead Cunningham:** Writing – review & editing, Writing – original draft.

## Funding

No funding was received

## Declaration of Competing Interest

The authors declare that they have no known competing financial interests or personal relationships that could have appeared to influence the work reported in this paper.
